# The Epidemiology of Syringomyelia and Associated Conditions: A Literature Review

**DOI:** 10.3390/jcm15218653

**Published:** 2026-11-06

**Authors:** Enver I. Bogdanov, John D. Heiss

**Affiliations:** 1 Department of Neurology, Kazan State Medical University, 420012 Kazan, Russia; 2 Surgical Neurology Branch, National Institute of Neurological Disorders and Stroke, National Institutes of Health, Bethesda, MD 20892, USA

**Keywords:** syringomyelia, syringomyelia epidemiology, syringomyelia prevalence, syringomyelia incidence, idiopathic syringomyelia, hydromyelia, dilated central canal, presyrinx, postsyrinx, Chiari I malformation

## Abstract

**Background/Objectives**: Syringomyelia is an intramedullary, fluid-filled cavity that expands the spinal cord, usually secondary to impaired cerebrospinal fluid (CSF) flow at the craniovertebral junction or spinal canal, or to spinal cord tethering. Its epidemiology is difficult to define because related entities—including hydromyelia, persistent or dilated central canal, slit-like cavities, presyrinx, postsyrinx, myelomalacia, glioependymal cysts, and cystic tumors—are inconsistently classified. This review evaluated syringomyelia classification and prevalence in relation to associated disorders and diagnostic criteria. **Methods**: A MEDLINE/PubMed and Google Scholar search was performed through May 2026 using terms related to syringomyelia, hydromyelia, central canal dilatation, slit-like cavities, presyrinx/postsyrinx states, Chiari malformations, prevalence, incidence, and classification. Eligible sources included clinical studies, cohort and case–control studies, family/genetic studies, reviews, historical articles, and book chapters. Duplicates and inaccessible full-text sources were excluded. **Results:** Since MRI became the diagnostic standard, syringomyelia has been detected earlier and more often, including in oligosymptomatic and asymptomatic patients. MRI has also improved identification of associated conditions, particularly Chiari malformation type 1 (CM1), basilar invagination, spinal pathology, and CSF pathway obstruction. Reported MRI-era prevalence varies substantially across populations, from 1.94 per 100,000 in Japan to 4.84 per 100,000 in Northwestern Italy and 8.2 per 100,000 in New Zealand, with higher rates in selected Russian regions with familial or ethnic clustering of CM1. CM1 is consistently the most common associated condition. However, prevalence estimates are affected by whether hydromyelia, persistent central canal variants, slit-like cavities, and presumed idiopathic syringomyelia are included. Apparent idiopathic cases may reflect occult CSF obstruction, arachnoid adhesions, Chiari type 0, or spinal canal abnormalities. **Conclusions**: Syringomyelia prevalence varies by geography, ethnicity, associated conditions, imaging era, and diagnostic definitions. Standardized clinicoradiological criteria are needed to distinguish true syringomyelia from related cavity conditions and improve epidemiological accuracy.

## 1. Introduction

Syringomyelia is an intramedullary, fluid-filled cavity that distends the spinal cord. The term syringomyelia (Greek: syrinx—pipe, myelos—marrow) was coined by Ollivier d’Angers in 1824, although the first observation of cavitation within the spinal cord in a cadaver dates to the 16th century [1]. Several anatomical forms, often related to syringomyelic cavitation, have been identified, including communicating, partial communicating, and noncommunicating syringomyelia with the fourth ventricle, central canal dilatations, hydromyelia, extracanalicular parenchymal cavitations, and atrophic cavitations. Some of these terms are used interchangeably; others remain disputed [2,3,4,5,6,7,8,9].

The remnant central canal, which may or may not be excessively dilated, appears on MRI as a filiform or slit-like cavity. There is currently no widely accepted criterion for differentiating an enlarged central canal from other intramedullary cavities. Some radiologists refer to central canal enlargement as syringomyelia, hydromyelia, or idiopathic syringomyelia. However, others do not classify this usually benign condition as syringomyelia [6,7]. This inconsistency in diagnostic terminology may reduce the accuracy of studies on syringomyelia prevalence and incidence. Along with syringomyelic cavitation, other intramedullary cavity-forming conditions have been identified, such as myelomalacia, glioependymal cyst, and cystic tumor [7]. These cavities are unrelated to syringes associated with CSF obstruction or spinal cord tethering and must be differentiated from them, which can be difficult in some cases. Including all types of intramedullary cavities under the diagnosis of syringomyelia may interfere with epidemiological studies of syringomyelia associated with CSF obstruction or spinal cord tethering.

Syringomyelic cavitations filled with CSF-like fluid do not usually arise from diseases within the spinal cord. Instead, they arise secondary to an underlying condition that obstructs cerebrospinal fluid (CSF) flow at the craniovertebral junction (CVJ) or within the spinal canal, or that tethers the spinal cord [5]. Some types of cavitation share similar mechanisms of formation, while others differ significantly. The pathophysiology of syringomyelia remains a topic of debate and is thoroughly discussed in other publications, which is beyond the scope of this review [5,10,11,12,13,14]. Interpreting results from epidemiological studies on syringomyelia is closely tied to how syringomyelia and related conditions are defined, as well as to the prevalence of associated underlying diseases. There is a notable lack of studies specifically focused on the epidemiology of syringomyelia itself, although several aspects of syringomyelia’s epidemiology have been examined in reviews concerning Chiari type 1 malformation (CM1) [15,16,17].

We used previously published definitions of prevalence and incidence in selected clinical populations when comparing epidemiological findings [15]. The incidence rate is the number of new cases per unit of person-time at risk. Cumulative incidence is the proportion of people who develop the condition during a specified period. Period prevalence is the proportion of individuals in a stable population who have the condition during a specific period. Point prevalence is the proportion of individuals in a population who have the condition at a specific time. Lifetime prevalence is the proportion of individuals who have had the condition at any time, including current cases and those previously diagnosed and treated [15].

## 2. Materials and Methods

This study was conducted as a narrative review with a structured literature search because the literature on the epidemiology of syringomyelia and related conditions across regions and time periods is heterogeneous. We conducted a bibliographic search in PubMed, Scopus, and Google Scholar. In addition, we manually searched the reference lists of included studies to identify other relevant studies. No language restrictions were applied. Selected non-English publications from before 1990 were obtained from the library of Kazan State Medical University. The studies ultimately included were research articles, original articles, books, and clinical cases that provided information on epidemiology and associated conditions. We examined the title, abstract, and keywords of each study and applied the inclusion and exclusion criteria. For potentially eligible studies, the same procedure was applied. Articles published between 1899 and 15 May 2026 were included. Inclusion criteria comprised studies (case series, retrospective cohorts, prospective studies, comparative studies, reviews, and classifications) presenting epidemiological data on different types of syringomyelia and related cavitary conditions of the spinal cord. Exclusion criteria comprised studies focused on syringomyelia in animals, studies for which the full text was unavailable, and letters to the editor. The following search string was used: (syringomyelia OR syrinx OR hydromyelia OR syringohydromyelia OR syringobulbia) AND (classification OR primary OR secondary OR idiopathic OR unknown etiology OR noncommunicating OR communicating OR canalicular OR extracanalicular) AND (remnant central canal OR dilated central canal OR persistent central canal OR slitlike OR slit-like OR incidentally identified OR presyrinx OR postsyrinx) AND (epidemiology OR prevalence OR incidence OR frequency OR distribution OR ethnic OR regional). A total of 1028 sources were identified. Subsequent screening removed duplicate publications and those lacking sufficient information to assess their relevance. As a result, 145 publications were selected, including prospective (n = 19), retrospective (n = 25), and cohort (n = 45) studies; family and genetic studies (n = 9); case–control studies (n = 26); and reviews and discussions (n = 8). Pertinent historical articles (n = 2) and book chapters (n = 11) provided additional information and perspective for the review. Due to substantial heterogeneity across study populations, interventions, and reported outcomes, a quantitative meta-analysis was not conducted.

## 3. Results and Discussion

### 3.1. The Frequency of Syringomyelia Related to an Underlying Disease

In a New Zealand study of 137 patients diagnosed with syringomyelia between 1961 and 2003, syringomyelia was associated with the following underlying conditions: CM1 in 64.3%, idiopathic (no other abnormality) in 16.1%, post-traumatic arachnoiditis in 9.5%, non-traumatic arachnoiditis in 4.4%, other posterior fossa abnormality in 2.9%, and isolated spinal cord abnormality in 2.9% [18].

In a nationwide survey of patients with syringomyelia in Japan, syringomyelia was associated with the following underlying conditions: CM1 in 48%, idiopathic in 15.7%, CM type 2 in 8.1%, trauma-related in 7.5%, spinal cord tumor in 5.2%, arachnoiditis in 4.9%, bone anomaly in 1.0%, and other forms in 7.9% [19].

In Roser’s and colleagues’ study of 142 patients with syringomyelia, neuroradiological evaluation identified CM1 in 26%, trauma in 19%, tumors in 12%, scoliosis in 5%, and meningitis in 2%. Idiopathic syringomyelia was identified in 36%, and 80% of idiopathic cases were classified as “hydromyelia” based on specific clinical, electrophysiological, and radiological criteria. Patients with hydromyelia had nonspecific pain syndromes and lacked neurological and electrophysiological abnormalities. Radiologically, hydromyelia was characterized by an intramedullary, centrally located, slit-like cavity. Intraspinal pathology did not disrupt CSF flow [20].

A study of 271 pediatric patients with a syrinx at least 3 mm in maximum axial dimension found that CM1-related (43.2%) and idiopathic (30.6%) were the most common associated conditions, followed by spinal dysraphism (7.4%), tumor (5.5%), tethered cord (4.8%), secondary Chiari malformation caused by craniostenosis (4.8%), and Chiari malformation type 0 (1.5%) [21].

A recent study classified syringomyelia as CM1-related (59%), pure hydromyelia (18%), secondary syringomyelia (14%), and idiopathic (9%) [22]. The authors defined “pure hydromyelia” as developmental widening of the spinal cord’s central canal [3].

Another study, using a broader list of predisposing conditions, found that idiopathic syringomyelia accounted for less than 5% of cases [23].

An analysis of the results from the five studies above indicates that CM1 is the condition most frequently associated with syringomyelia across all studies. In most studies, idiopathic syringomyelia was the second most frequent form. The high prevalence of idiopathic syringomyelia may be exaggerated, as asymptomatic individuals with persistent central canals may have been included as cases of idiopathic syringomyelia. Using the terms syringomyelia and hydromyelia to describe asymptomatic individuals with a dilated central canal can inflate the proportion of patients characterized as having syringomyelia or hydromyelia. A more detailed clinical and radiological approach is needed to nosologically differentiate entities such as hydromyelia, persistent central canal, a slit-like cavity, and idiopathic syringomyelia.

### 3.2. Records of Syringomyelia Prevalence in the Pre-MRI Era

In the pre-MRI era, clinicians diagnosed syringomyelia based on clinical grounds alone. Following the classical description of its neurological presentation, signs and symptoms of slowly progressive central myelopathy alerted clinicians to syringomyelia. Neurological deterioration was more rapid with intramedullary neoplasms than with syringomyelia. Syringomyelia is often described as a crippling neurological disorder [24,25,26,27,28]. In Europe, from the late 19th century to the first half of the 20th century, syringomyelia was among the most common spinal cord diseases [28]. An analysis of 11,800 inpatient medical records from the neurological department of Kazan State Medical University for the periods 1937–1941 and 1945–1962 found that 7.3% of inpatients were diagnosed with syringomyelia [29].

In the early and mid-20th centuries, individuals engaged in physically demanding work were more likely to develop syringomyelia [29,30]. From the 1940s to the 1970s, the prevalence of syringomyelia in Europe declined significantly [31,32,33,34]. The decline in incidence during the pre-MRI era may reflect fewer severely affected patients with syringomyelia, since diagnosis in that era depended on a typical time course of slowly progressive central myelopathy and an intramedullary lesion with a collapsing cord sign on myelography. Syringomyelia cases with milder central myelopathy were less likely to be diagnosed by clinical examination than cases with advanced myelopathy. One could speculate that before 1940, physical labor was more strenuous than after 1940, leading to more frequent heavy straining and increases in intraspinal pressure, which exacerbated asymptomatic and subclinical forms of syringomyelia and converted mild forms into more severe ones. In 1958, in Rochester, Minnesota (USA), a population of 30,000 had only one patient with syringomyelia. The authors estimated the prevalence of syringomyelia at 3.3 per 100,000 [35]. Notably, in the USA in the mid-1950s, classical syringomyelia was considered an uncommon disease of the neuraxis [36]. In 1963, in Iceland, with a population of 187,200 at the time, there were 19 patients with syringomyelia; the prevalence rate was 10.14:100,000, with an incidence of 0.29:100,000 per year [37]. In an English city in 1966, the prevalence rate of syringomyelia was 8.4:100,000 (6 cases per 70 000 inhabitants) [38]. An interesting observation is that in the state of Mississippi (USA), syringomyelia was more common in African Americans than in Caucasians [39]. In the pre-MRI era, the incidence of syringomyelia was higher in the Lower Franconia and northern Baden areas of Germany (0.43 and 0.44 per 100,000 per year) than in the northern and southern areas of Württemberg (0.16 and 0.09 per 100,000 inhabitants per year) [32].

In the Russian Federation, in Gorky province (Nizhny Novgorod), during the pre-MRI era, the prevalence of syringomyelia ranged from 0.3 to 5.6:100,000, with a mean of 2.4:100,000. Some Gorky province settlements with familial syringomyelia cases showed the high end of this range [40]. In Tatarstan, a Russian Federation republic roughly 320 km away, the mean prevalence of syringomyelia in the pre-MRI era was similar to that of Gorky province, at 2.8:100,000 [41]. In Bashkortostan, neighboring Tatarstan, 1258 cases of syringomyelia were identified in the pre-MRI era. These studies showed that prevalence varied widely across regions, ranging from 2.9 to 98 per 100,000 [42]. The authors highlighted the marked uneven distribution among ethnic groups in this region, with higher prevalence among Tatars [30,42]. Interestingly, the prevalence of syringomyelia in Uzbekistan, a part of the Soviet Union until 1991, was significantly lower than in other Soviet regions. An analysis of medical data from 25,040 neurological inpatients in the Samarkand district in southeastern Uzbekistan, covering 1930 to 1970, identified only 97 patients (0.38% of all hospitalized patients) with syringomyelia. Notably, the 97 patients with syringomyelia were mainly hard physical workers, and most (72%) were not indigenous to the area [33].

In summary, most countries with predominantly Caucasian populations during the pre-MRI era reported syringomyelia prevalence ranging from 0.3 to 8.5:100,000. However, syringomyelia prevalence was significantly higher in some settlements with Tatar populations in the Volga-Ural region of the Soviet Union, now the Russian Federation [30,42]. This range of syringomyelia prevalence is commonly cited in the current literature and is considered fundamental. However, in the pre-MRI era, diagnostic radiologic tests for syringomyelia were less sensitive and specific than MRI. In addition, the prevalence recorded in the pre-MRI era consisted of patients with the classically manifested severe form of syringomyelia, diagnosed late in the course of their disease and often without surgical treatment. These factors led to a lack of recognition of asymptomatic, oligosymptomatic, or stable syringomyelia cases.

### 3.3. Records of Syringomyelia Prevalence in the Post-MRI Era

Since the MRI era began in 1985, MRI has been the gold standard for diagnosing syringomyelia. As a result, syringomyelia is diagnosed earlier, before spinal cord dysfunction reaches an advanced stage. MRI has also improved the diagnosis of conditions associated with syringomyelia, such as Chiari I malformation, basilar invagination, and spinal pathology. Obstruction of CSF pathways is a factor associated with syringomyelia. MRI can document relief of CSF pathway obstruction and reduced syrinx size after successful surgery. Currently, clinicians diagnose and treat symptomatic syringomyelia earlier, reducing the number of debilitating cases. Syringes are occasionally found in asymptomatic subjects. Enlargement of the central canal of the spinal cord is not syringomyelia. Patients with central canal enlargement do not have a neurological deficit but may have pain in a region that does not correlate with the involved cord levels. More cases of syringomyelia are detected in the MRI era than before. However, the prevalence of syringomyelia is still underestimated, although to a lesser extent, in the MRI era because not everyone undergoes MRI scanning to detect syringomyelia.

Between 1985 and 1989, the prevalence of syringomyelia among eighteen-year-old male recruits in the central provinces of Spain was 0.9 per 100,000 [43]. In 2003, the prevalence of syringomyelia in the entire population of New Zealand was 8.2 per 100,000. Among New Zealand Caucasians, who comprised 66% of the population in 2001, the prevalence was lower, at 5.4 per 100,000. In other ethnic groups, the prevalence was significantly higher. Among Pacific people and the Māori, the prevalence was 18.4 and 15.4 per 100, 000, respectively. Syringomyelia was more often associated with CM1 in Pacific people (84.4%) than in the Māori (42.9%) or in other Caucasians (38.2%) [18].

In the Bashkortostan Republic of the Russian Federation, a study of more than 2000 syringomyelia cases found a wide range in prevalence, from 3.5 to 129:100,000. Ethnic Tatars had a higher prevalence of syringomyelia [44]. In Bashkortostan, CM1 accounted for 72% of syringomyelia cases [45]. Tatarstan, located west of the Bashkortostan Republic in the Russian Federation, had an estimated mean syringomyelia prevalence of 12.6:100,000 in 2011 [46]. This MRI-era syringomyelia prevalence is more than 4 times the pre-MRI era prevalence reported for the same region. In a district of Tatarstan with a very high prevalence of Chiari type 1 malformation (CM1), 413 per 100,000, the incidence of syringomyelia was also elevated, with about one-half of CM1 patients also having MRI-verified syringomyelia [47].

Evidence on the prevalence and incidence of syringomyelia in Western Europe is limited. A study reported that in 2011 in Northwestern Italy, the prevalence of syringomyelia was 4.84:100,000, the CM1 prevalence was 7.74:100,000, the syringomyelia incidence was 0.82:100,000, and the CM1 incidence was 3.08:100,000 [22].

A nationwide survey in Japan estimated the prevalence of all etiological forms of syringomyelia to be substantially lower, at 1.94:100,000, than in Western and Eastern Europe and New Zealand [18]. In Japan, 48% of syringomyelia cases were associated with CM1 [19]. The lower prevalence of syringomyelia in Japan than in Europe aligns with previous data indicating that the prevalence of tonsillar ectopia is lower in Japan (0.24%) than in the Euro-American population (2–5%) [48,49,50].

In summary, the prevalence of syringomyelia in the European population was estimated at 0.9–11:100,000 in both the pre- and post-MRI eras. However, the clinical profile of patients with syringomyelia differs between these two eras. At the end of the 19th century and in the first half of the 20th century, patients presented with classical manifestations of syringomyelia (dissociated sensory loss with preservation of tactile sensation and loss of temperature and pain sensation, hand and arm weakness, gait spasticity) at an advanced stage of the disease, with substantial neurological deficit. From the end of the 20th century to the present, a significant proportion of patients with syringomyelia are oligo- or asymptomatic. Syringomyelia prevalence is higher when idiopathic syringomyelia includes MRI-evident enlarged central canals or slit-like cavities with nonspecific symptoms. Past reports of syringomyelia clustering in some areas of the world were confirmed in the post-MRI era, when CM1-related ethnic and familial clusters of syringomyelia were described [46,47,51].

### 3.4. Characterization of Syringomyelia in Relation to the Interpretation of Epidemiological Records

Definitions, classifications, and terminology related to syringomyelia are highly controversial. Syringomyelia is generally defined as a polyetiological neurological disorder characterized by the development of an expansive, fluid-filled, longitudinally oriented cavity (syrinx) within the spinal cord parenchyma. Syringomyelic cavities are median (canalicular), paramedian (extracanalicular), or combined intra- and extracanalicular parenchymal, fluid-filled formations. They most commonly originate in the cervical or cervicothoracic region but can extend both upward and downward.

Initially, syringomyelia communicating with the fourth ventricle through the central canal was termed hydromyelia. The term syringomyelia was reserved for cysts that did not communicate with the fourth ventricle through the central canal. With the widespread use of MRI, it became clear that only a minority of syrinxes communicated directly with the fourth ventricle, blurring the theoretical distinction between hydromyelia and syringomyelia. Both terms began to be used interchangeably, and combined terms, such as syringohydromyelia or hydrosyringomyelia, were also introduced.

On MRI, the central canal can appear as a slit-like spinal cavity and may be mislabeled as syringomyelia [52]. Roser and colleagues reported that hydromyelia, defined as a dilated spinal canal, is not a disease with an underlying pathology and does not exhibit the clinical, electrophysiological, or radiological characteristics of patients with true syringomyelia [20]. The medical literature refers to slit-like centromedullary cavities as hydromyelia, dilated central canal, and sometimes idiopathic syringomyelia [7,20,52,53,54,55,56,57,58]. These slit-like, filiform, fusiform, “localized” or “extended” spinal cord cavities represent a persistent central canal that is normally present at the thoracic or cervical level and can measure 1–4 mm in diameter in 1.5% of adults without underlying pathology [6,52,54,56,57,59]. Enlargement of the central canal in the conus medullaris, also known as the ventriculus terminalis, is a common finding on MRI scans of infants and is typically considered unrelated to syringomyelia [60]. Although a residual central canal could enable another process to form an expanded true syrinx cavity [61], it is usually a normal variant that does not cause symptoms [6]. Syringomyelia occurs in patients with an underlying pathology.

**Figure 1:**
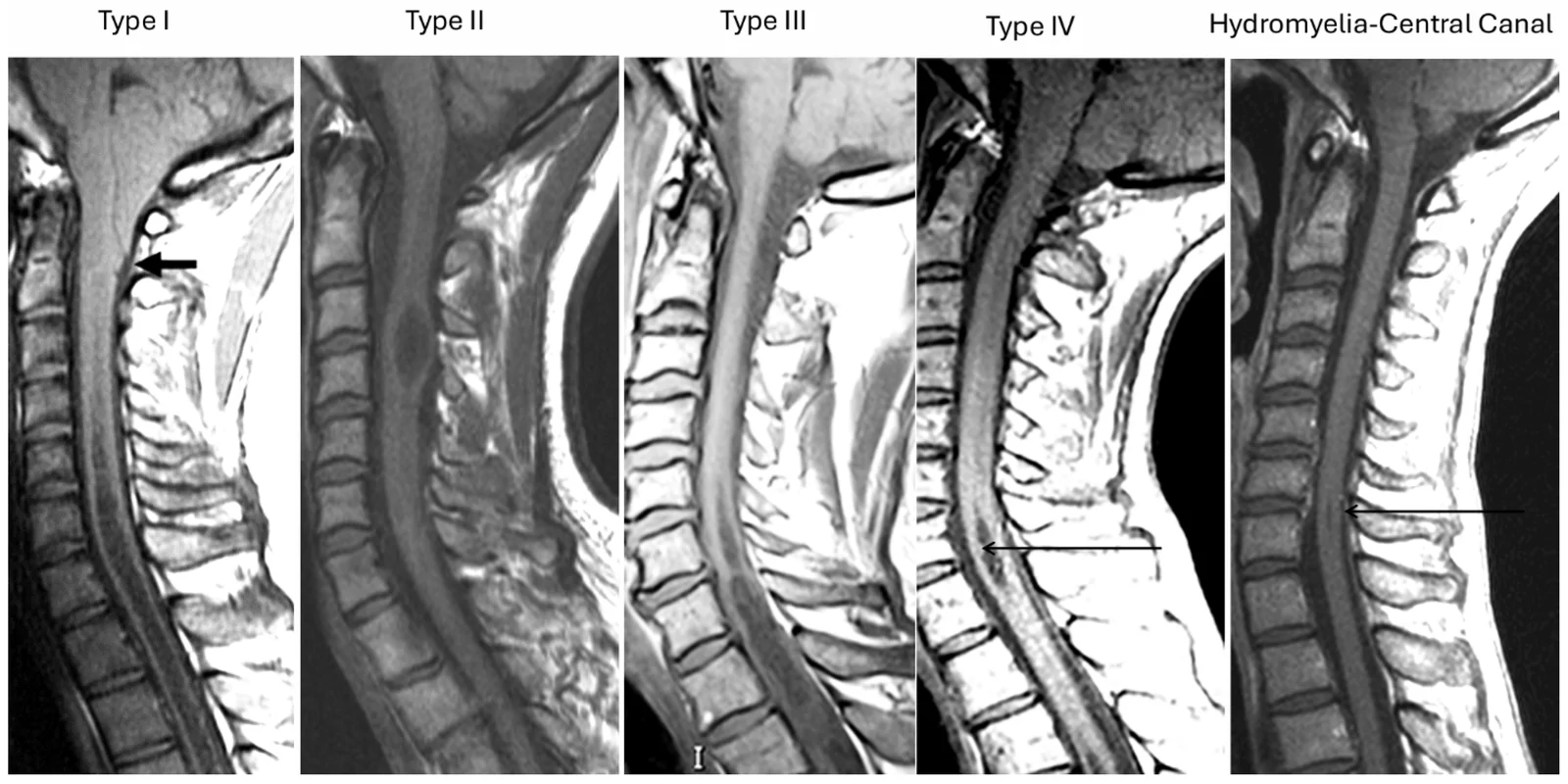
Radiological differences among syringomyelia, hydromyelia, persistent central canal, and idiopathic syringomyelia, based on the classification and descriptions in the Consensus Document [3]. The bold arrow (leftmost panel) indicates the inferior extent of the cerebellar tonsils in a patient with Type I Chiari malformation and syringomyelia. The narrow arrow (second panel from the right) points to a syrinx, and the narrowest arrow (rightmost panel) points to a slit-like cavity representing the spinal cord’s central canal.

Thin, central intramedullary fluid collections are referred to as hydromyelia, idiopathic syringomyelia, and dilated central canal. Some clinicians use these terms interchangeably; others consider them distinct entities. More than half of patients with hydromyelia, dilated central canal, or idiopathic syringomyelia present with pain or dysesthesia of the limbs; 10–44% have minor neurological deficits, and about a third are asymptomatic. In most cases, symptomatic patients are clinically stable or have improved [7,20,52,53,54,55,56,57]. Some metrics have been proposed in pediatric patients to distinguish a dilated central canal from a narrow syrinx on imaging. Syringomyelia is defined as fluid cavities exceeding 1–2 mm in axial dimension and extending at least 2 vertebral levels on MRI [56,57]. Magge and coauthors noted no strong correlation between cavity size and the presence or severity of symptoms [56]. In another pediatric study, 98 of 275 (35.6%) patients with syringomyelia were classified as idiopathic “syringohydromyelia,” with a median diameter of 2 mm [62]. Fewer than half of patients presented with any neurological deficit, and 38% had concomitant scoliosis. Strahle and coauthors speculated that the wide CM1–associated syringes may cause scoliosis, whereas the thin “idiopathic” syringes may form because of the curve itself. In pediatric patients, Strahle and coauthors used a 3 mm cavity width as a minimum threshold for diagnosing syringomyelia, thereby excluding cases of very thin central spinal fluid collections within the central canal [21]. A consensus conference defined pure hydromyelia as “developmental widening of the central canal of the spinal cord” and classified it as the Type IV class of “Syringomyelia-hydromyelia” [3]. In contrast, hydromyelia-central canal was not classified as a type of syringomyelia and was defined as “an intramedullary, centrally located, non-enhancing, slit-like cavitation, often confined to a short segment of the spinal cord and occurring in a non-enlarged or only slightly enlarged spinal cord (idiopathic localized hydromyelia) not associated with clinical symptoms” [3]. These definitions recognize that pure hydromyelia and hydromyelia with central canal dilatation are distinct entities. Table 1 and Figure 1 summarize the distinguishing characteristics of syringomyelia, hydromyelia, persistent central canal, and idiopathic syringomyelia, as outlined in the Consensus Document [3].

Including pure hydromyelia as a class of syringomyelia-hydromyelia in that consensus conference could increase the reported prevalence of syringomyelia in epidemiologic studies. Another approach to classifying syringomyelia would require an association with an underlying disorder and exclude intramedullary fluid collections without underlying pathology.

A dilated central canal or hydromyelia, as identified on radiologic imaging, can co-occur with CM1 and other extramedullary and intramedullary conditions. The dilated central canal, often appearing as a slit-like cavity, is commonly found in both symptomatic and benign cases of CM1 [63,64]. Additionally, nearly 5% of symptomatic pediatric patients with CM1 have isolated thoracic syringes that resemble a dilated central canal [65]. In CM1, the prognostic significance of MRI-detected dilated central canal remains unknown. Some dilated central canals may develop into syringes over time.

Idiopathic syringomyelia is a condition in which no overt etiology is evident for a syrinx [58]. Distinguishing idiopathic syringomyelia from central canal dilatation, which typically lacks an underlying pathology, is particularly challenging [7,21]. In any cohort of patients with syringes, it is important to distinguish those with a dilated central canal from those with thin syringes. According to Strahle and coauthors, a width of 5 mm or less, combined with a cranial extent in the thoracic or lumbar spine, has high specificity for idiopathic syrinx. This finding may help determine causation when evaluating a patient with syringomyelia [21]. All patients with idiopathic syringomyelia should be carefully examined. In one study, 39% (125 of 320) of syringomyelia patients were diagnosed with idiopathic syringomyelia. In 26% (33 of 125) of these patients, the underlying cause was identified (cardiac-gated phase-contrast MRI of CSF flow showed CSF flow blockage). The other 74% of patients had no identified underlying cause of syringomyelia (CSF flow was unobstructed) [66]. In another study of 10 patients with apparent idiopathic syringomyelia, CT myelography revealed CSF blockage due to adhesions of an arachnoid web in 9 of them [67].

Patients with a preliminary diagnosis of idiopathic syringomyelia at the cervical level may show radiologic features of a small posterior cranial fossa, typical of CM1 [68,69]. In these cases, syringomyelia results from Chiari malformation type 0 (CM0) [3]. In CM0, posterior fossa crowding, CSF obstruction, or foramen magnum arachnoiditis, rather than absolute tonsillar descent, are the principal drivers of syringomyelia development, including cases that appear idiopathic [70,71]. These cases of apparent idiopathic syringomyelia show increased peak CSF flow velocity at the foramen magnum, confirming disruption of normal CSF flow [72]. Patients with idiopathic syringomyelia may have abnormally narrow upper and mid-cervical canal diameters, which can alter CSF flow dynamics and contribute to syringomyelia [73]. Thus, the primary condition underlying syringomyelia in cases initially diagnosed as idiopathic appears to align with Klekamp’s reasoning [71], who questions whether a true idiopathic syrinx exists. Interestingly, a dilated central canal (hydromyelia) may be reversible [74,75,76]. A synchronized swimmer with recurrent thoracic girdle pain was described, and MRI detected CM1 and hydromyelia. The swimmer followed medical advice to avoid straining or holding her breath. The pain resolved over the next 10 days. Follow-up MRI of the thoracic cord showed the absence of the initial abnormal finding [74].

Some conditions can mimic a dilated central canal. The anterior median fissure of the spinal cord, which resembles the central canal, is present in some healthy individuals and in CM1 patients. It is not the central canal or hydromyelia and should be distinguished from them. This differentiation is important for accurate MRI diagnosis and for determining the prevalence of dilated central canal, given the high incidence of radiologically visible hyperintense foci in the anterior median fissure of the spinal cord [77].

In Milhorat’s classification, atrophic cavitations, defined as microcysts, intramedullary clefts, and localized dilations of the central spinal canal, were identified separately. These cavitations develop in the context of degenerative changes in the spinal cord that accompany spinal cord atrophy. Atrophic cavitations caused by loss of parenchymal tissue (syringomyelia ex vacuo) do not propagate, presumably because of the absence of an active filling mechanism [8]. Strahle and colleagues speculated that idiopathic syringes may arise from stretching of the canal by the denticulate ligaments, with passive accumulation of fluid in the spinal cord [21]. Several primary degenerative disorders associated with spinal cord atrophy and central canal dilatation have been described: Charcot-Marie-Tooth disease, hereditary spastic paraplegia, and amyotrophic lateral sclerosis [78,79,80]. The true prevalence of syringomyelia ex vacuo is unknown.

Accumulated data on radiologically identified stages of syringomyelia development may inform diagnostic and epidemiological studies. Several authors have described longitudinal spinal cord edema that preceded syrinx formation at the same location and termed it “presyrinx syndrome”. Presyrinx can present as an acute or chronic myelopathy or be asymptomatic. Symptoms of presyrinx and associated radiologic findings may be reversible with correction of CSF obstruction [81,82,83,84,85].

Almost every patient with symptomatic syringomyelia experiences progression of neurological symptoms over time, either gradually or in steps. However, the rate of neurological deterioration varies [71]. In long-standing syringomyelia, MRI studies may show that the syrinx collapses, leaving the spinal cord atrophic rather than distended [8]. In a study of patients with untreated symptomatic syringomyelia lasting over 20 years, syrinx diameter was significantly smaller in those with shorter disease duration [86]. In some cases, a slit-like cavity represents the late phase of spontaneous resolution of a preexisting, larger syrinx in CM0 or CM1 and does not belong to the dilated central canal subtype, which assumes no underlying condition [87,88].

A syrinx can spontaneously collapse, halting clinical deterioration. This may result from the natural resolution of the CSF blockage, disruption of the syrinx walls, or reduced daily physical strain [89,90,91]. Some patients may experience residual symptoms known as “postsyrinx syndrome” [87,88,92]. The true incidence and prevalence of “presyrinx” and “postsyrinx” syndromes, with or without syringomyelia-like manifestations, remain unknown.

Hydromyelia, dilated central canal, and idiopathic syringomyelia are difficult to distinguish as separate conditions. However, accurate classification of these disorders is important for identifying the specific abnormality, developing a classification system, and determining appropriate treatment and prognosis. Technical advances in imaging and electrophysiology will help identify slit-like cavities and their associated neurological outcomes. The main features distinguishing central canal dilatation from idiopathic syringomyelia were described, and a management algorithm for patients with idiopathic syringomyelia was proposed [61,93].

### 3.5. Epidemiology of Chiari Malformation Type 1 and Craniocervical Junction-Related Syringomyelia

Chiari malformation type 1 (CM1) is the most common underlying cause of syringomyelia and disrupts CSF flow at the CVJ. CM1 is traditionally defined as cerebellar tonsillar position 5 mm or more below the plane of the foramen magnum (FM) on midsagittal MRI [94,95,96]. Tonsillar position between 3 mm and 5 mm is considered borderline for CM1 and abnormal only if associated with syringomyelia or peg-like tonsils [3]. CM1 encompasses a diverse range of disorders with various pathogenic origins. Morphometric analysis of the PCF is required to distinguish Primary (classical) CM1, occurring with a pathologically small PCF, from Secondary (acquired) CM1, in which the PCF size and volume are normal and tonsillar herniation is caused by other mechanisms, such as intracranial hypertension, intraspinal hypotension, spinal cord tethering, cranial setting, CVJ instability, or secondarily reduced skull volume in craniosynostosis [94,97]. In the current Consensus Document, Secondary CM1 is termed “acquired tonsillar ectopia.” Primary CM1 is defined as a “paraxial mesoderm disorder, with abnormalities of the posterior cranial fossa (mostly small) and the consequent herniation of the cerebellar tonsils: CM1-A: with syringomyelia on MRI; CM1-B: without syringomyelia on MRI” [3].

Primary CM1 is the most common form of CM1 [94]. A recently proposed classification of CM1 is based on the mechanisms causing ptosis of the brainstem and cerebellum, as described by Milhorat and coauthors [98,99,100]. Within that framework, morphometric assessments of the PCF differentiate primary CM1 subtypes from secondary CM1 [94,98,100].

Current data suggest that CM1 is not a rare disorder, but the exact prevalence of CM1 remains unknown, according to 93.2% of experts [101]. The comprehensive analysis demonstrated that the prevalence of CM1 ranges from 0.5% to 3.5% in the general population, from 0.56% to 0.77% in MRI studies, and from 0.62% to 0.77% in anatomical brain-dissection studies [102]. Regional and familial clustering of CM1 has been described, with significantly higher prevalence in Tatarstan and Bashkortostan of the Russian Federation [44,46,47] and in the state of Utah in the USA [51]. Accumulated data show that the prevalence of primary CM1, which develops due to a small posterior cranial fossa, depends substantially on genetic factors that determine syringomyelia prevalence. In 12% of symptomatic patients with classical CM1, a positive family history of CM1 was found [96]. Several PCF traits in CM1-affected families are heritable, and more than half are associated with disease status. PCF volume, basal angulation, and height are highly heritable, whereas tonsillar herniation that blocks CSF flow and causes syringomyelia is influenced by environmental factors [103,104].

The prevalence of CM1 diagnosed by radiographic criteria is considerably higher than that of symptomatic CM1 [15,105]. Radiographically identified CM1-related syringomyelia can also be asymptomatic, even when the syrinx axial diameter is 8.5–9.0 mm [106]. In one study, 38% of patients with CM1-associated syringomyelia were asymptomatic [22].

In healthy, asymptomatic adults who volunteered for MRI, an incidental CM1, defined by tonsillar position, was identified in 0.9% [107]. In a database of randomly selected patients aged 21–50 who underwent consecutive MRI, tonsillar descent of 5 mm or more was detected in about 2% [50].

A lower tonsil position is linked to a higher likelihood of CM1 symptoms and syringomyelia. However, the association between tonsillar herniation (TH) and symptom severity is weak, with many individuals having TH of 5–15 mm yet remaining asymptomatic [95]. Cases of minimal or subthreshold (<5 mm) TH can be symptomatic [108]. When these patients have syringomyelia, the condition is classified as Chiari Type 0 malformation (CM0) [3,70,109,110]. As a result, 88.5% of experts agreed to reject the 5 mm rule for diagnosing CM1 [101]. Greater TH is associated with more severe alterations in CSF flow at the foramen magnum and within the spinal syrinx [111,112].

The Primary CM1 type encompasses a continuum of subtypes, including CM0, CM0.5, CM1, and CM1.5; each may be causative for syringomyelia [99,109,110,113]. Syringomyelia is considered an obligatory sign of CM0 [110]. Currently, CM0 is defined as “obliteration of the cisterna magna (due to arachnoid adhesions) and/or a volumetrically small posterior fossa, with cerebellar tonsils positioned at the foramen magnum and a syringomyelia in the cervical spinal cord” [3]. CM1 experts consider narrower syrinxes typical of CM0 [101]. A subsequent study confirmed that mean syrinx size was smaller in CM0 patients than in CM1 patients [70].

CM 0.5 is defined as a ventrolateral herniation of the cerebellar tonsils anterior to a horizontal line drawn through the midpoint of the caudal medulla at the foramen magnum [110]. Patients with CM 0.5 are more likely to present with medullary symptoms. Of 11 pediatric patients in a surgical CM 0.5 series, 4 (36%) had syringomyelia [114].

CM1.5 malformation is a more severe form of CM1, characterized by greater crowding of the foramen magnum by brain tissue. CM1.5 is also characterized by ectopia of the medulla and cerebellar tonsils. Patients with CM1.5 malformations have symptoms and signs typical of Primary CM1 subtypes, although they are slightly more likely to develop severe symptoms, such as bulbar signs. Syringomyelia develops in 50–81% of patients with CM1.5 [109,115,116,117].

CM1 is the leading cause of syringomyelia. In northern Italy, among 436 patients with syringomyelia, CM1-related syringomyelia was identified in 59%; in the Republic of Bashkortostan of the Russian Federation, among 712 patients with syringomyelia, CM1-related syringomyelia was identified in 72% [22,45]. In a German surgical series of syringomyelia, 543 (35%) of 1535 patients had CM1-related syringomyelia [7]. The assumption that 70% of Chiari I patients have concurrent syringomyelia is a rough approximation in adults, with lower rates in pediatric studies. Reported proportions of syringomyelia cases due to CM1 are generally estimated at 51–80% in adults [47,96,118,119,120], 59% in adults and children [22], and 23% in children (particularly older children) [112]. Among CM1 patients selected for surgery, 69% of adults and 40% of pediatric patients had an associated syrinx [102]. Heiss and Argersinger estimated the prevalence of CM1-related syringomyelia at 5.9:100,000 by multiplying the prevalence of syringomyelia in an English city (8.4:100,000) in the pre-MRI era by the proportion of syringomyelia cases due to CM1 detected in the post-MRI era (about 70%) [15]. In the post-MRI era in northern Italy, CM1 prevalence was 7.74:100,000, and syringomyelia prevalence was 4.84:100,000 [22]. (Using a 70% estimate of the proportion of syrinx cases related to CM1 would have yielded a prevalence of CM1-related syringomyelia of 5.4:100,000 in northern Italy). In New Zealand, syringomyelia prevalence varied by ethnicity: among Pacific people, it was 18.4:100,000; among Māori, 15.4:100,000; and among Caucasians or others, 5.4:100,000 [18]. Using a 70% estimate for the proportion of CM1-related syringomyelia cases, the prevalence of CM1-related syringomyelia would be 12.9, 10.8, and 3.8 per 100,000, respectively, in these groups. If this method is applied to the Russian Federation, where syringomyelia is most prevalent in Bashkortostan at 129:100,000 [42], the prevalence of CM1-related syringomyelia would be 90:100,000. The prevalence of CM1-related syringomyelia was 0.81:100,000 in a California medical insurer’s database of MRI scans performed in children under 20 years of age over the previous 2 years [105].

Currently, basilar invagination (BI) is defined as a condition in which the tip of the dens extends more than 5 mm above the Chamberlain line [121]. BI is commonly cited as a cause of syringomyelia. However, no data are available on BI’s contribution to syringomyelia prevalence. BI may occur synchronously with CM1 or present as an isolated finding. The role of CM1 in syringomyelia development is well known, whereas BI’s contribution remains unclear. Data on the incidence of BI among patients with CM1 indicates a combined incidence of 12–24.6% in surgical series of CM1 patients with and without syringomyelia [96,122,123]. In 316 Chinese CM1 patients with syringomyelia, 23.4% had BI [124]. Notably, in the pre-MRI era in Europe, around one-third of syringomyelia patients demonstrated BI detected by X-ray [125]. In India, syringomyelia was identified in 50% of patients who had both BI and CM1, whereas patients with isolated BI had no syringomyelia [126]. Another study found syringomyelia in 57.1% of patients with BI without CM1 and 61.8% with a combination of CM1 and BI. In 34.7% of patients with BI and concomitant CM1, dilatation of the central canal without a space-occupying effect on the spinal cord was detected [127]. The author noted that these dilatations were considered evolving syrinx cavities [128] or as representing anatomical variations without clinical significance. In this study, syrinx cavities that showed a space-occupying effect before surgery decreased postoperatively, whereas foramen magnum decompression did not change the dilated central canal size [127]. The contribution of BI to the pathogenesis of syringomyelia in patients with coexisting BI and CM1 is supported by syrinx resolution after ventral decompression of the cervicomedullary junction and reestablishment of the CSF pathways [129]. The incidence of BI and CM1 together is expected to vary across regions. Evidence suggests a higher prevalence of BI in India and northeastern Brazil [130,131,132,133].

### 3.6. Epidemiology of Primary Spinal Syringomyelia

Primary spinal syringomyelia is syringomyelia that occurs in the absence of a foramen magnum abnormality but arises from spinal abnormalities [61]. In general, primary spinal syringomyelia accounts for about one-sixth of all syringomyelia cases [134]. This condition may arise from traumatic injuries, inflammatory conditions, or compressive lesions that disrupt the subarachnoid space. Syringomyelia can also occur when extramedullary tumors, osteophytes, or herniated intervertebral discs compress the CSF pathways. In contrast, when syringomyelia is associated with an intramedullary tumor, the CSF pathways are typically not obstructed, and the fluid within the syrinx is believed to originate from the tumor itself [10].

Posttraumatic syringomyelia accounts for 7.5–19% of all syringomyelia cases [18,19,20]. It is presumed to develop when the subarachnoid space is significantly narrowed or obliterated at the level of injury. Several subtypes of this syringomyelia have been described [11,61]. The incidence of syringomyelia after trauma has been estimated at 1–9%. If all patients with spinal injuries are investigated between 1 and 30 years after injury, 21–28% will be found to have a syrinx, and 30–50% will show some degree of spinal cystic change [135,136,137,138,139].

Inflammatory and post-inflammatory syringomyelia result from myelitis, demyelinating disorders, and intradural scarring from meningitis [61,140]. One study found that 4.5% of patients with multiple sclerosis exhibited MRI evidence of syringomyelia. However, the syrinx size was typically small, and these cavities appeared to be dilatations of the central canal [141,142]. Other demyelinating diseases have been reported to cause a reversible, slit-like cavity that results in central canal dilatation, referred to as non-communicating syringomyelia or hydromyelia [143]. Kim and colleagues reported that 7 (1.7%) patients in their 406-patient syringomyelia database had syringomyelia related to demyelinating disorders [23]. An MRI study of the spinal cord in patients with neuromyelitis optica and recurrent extensive myelitis detected cavitation in 16% of attacks [144]. Remnants of the central canal may serve as a nidus for the development of intra- and extracanalicular syringomyelia, consistent with Milhorat and colleagues’ hypothesis [6,145].

Syringomyelia progresses most rapidly in the early phases. High risk for progressive myelopathy is strongly predicted by concurrent Obstructive Sleep Apnea-Hypopnea Syndrome (OSAHS) and a positive Babinski sign; extensive cervicothoracic involvement; distended cavity morphology; and severe CSF pathway obstruction at the craniocervical junction [86,146]. Asymptomatic patients with small cervical syringes, no OSAHS, and a negative Babinski sign can be managed safely with periodic clinical and MRI follow-ups. Surgery is indicated for patients with progressive neurological deficits or those presenting with high-risk features (OSAHS, positive Babinski sign, and large cervicothoracic syringes) to prevent irreversible spinal cord injury [146]. Most incidentally discovered, small (≤3 mm), or asymptomatic syringes, including persistent central canal dilatations, follow a benign, static course and do not require surgery [7,52]. In long-standing unoperated cases, syringes may remain stable or spontaneously collapse as natural CSF flow is re-established [86,87].

## 4. Conclusions and Future Directions

Syringomyelia prevalence depends on the prevalence of conditions that predispose to syringomyelia and on the prevalence of syringomyelia without an associated condition, so-called idiopathic syringomyelia. The most common condition associated with syringomyelia is CM1. CM1-related syringomyelia accounts for 26–64% of cases. Idiopathic syringomyelia is the next most common form, accounting for 9–36% of cases. The prevalence of MRI-confirmed syringomyelia was 4.84:100,000 in Caucasians in Northwestern Italy and 5.4:100,000 in New Zealand [18,22]. In New Zealand, syringomyelia prevalence was higher in Pacific (18.4:100,000) and Māori people (15.4:100,000) than in Caucasians (5.4:100,000) [18]. In Japan, the prevalence of syringomyelia is significantly lower (1.9 per 100,000) than in Northwestern Italy and New Zealand [19]. The prevalence of syringomyelia may vary significantly across countries with large territories and multiethnic populations, particularly in regions where families or specific ethnic groups with CM1 are clustered. In the Russian Federation, the prevalence varies widely, from 0.32 to 130:100,000. CM1-related syringomyelia had higher prevalence in areas with familial clustering and in some predominantly ethnic Tatar settlements in the Volga-Ural region of the Russian Federation [44,46,47]. Data from key epidemiological studies of syringomyelia are presented in Table 2.

Estimates of syringomyelia prevalence that count cases with an enlarged central canal as syringomyelia can overstate prevalence. Epidemiological studies of syringomyelia prevalence struggle with their inclusion criteria. Studies must decide whether their definition of syringomyelia includes (1) developmental, significant widening of the central canal, designated as “pure hydromyelia,” which is mostly asymptomatic or presents with nonspecific symptoms, and (2) asymptomatic, non-progressive slit-like cavitation defined as “hydromyelia-central canal dilatation” or “enlarged central canal.” Radiologists often diagnose these as idiopathic syringomyelia. The remnant central canal cannot be termed syringomyelia if no filling mechanism is identified. The persistent central canal can serve as a nidus for syringomyelia in medullary or extramedullary settings [6,52,57,145]. A filiform or fusiform shape, smaller diameter, and absence of myelopathy can distinguish patients with these intramedullary CSF reservoirs from those with active syringomyelia that distends the spinal cord and causes myelopathy. However, a narrow syrinx can appear late in the course of syringomyelia, presumably after a distended syrinx deflates due to spontaneous resolution of Chiari I malformation or spontaneous syrinx drainage into the subarachnoid space [86,87,89,90,91]. Continued research is essential to accurately determine the prevalence and incidence of syringomyelia and other spinal cord cavity conditions worldwide.

## Figures and Tables

**Table 1:** Clinical and radiological differences among syringomyelia, hydromyelia, persistent central canal, and idiopathic syringomyelia, based on the classification and descriptions in the Consensus Document [3].

Type of Syringomyelia-Hydromyelia	Syringomyelia (Type I): Secondary to Foramen Magnum Obstruction	Idiopathic Syringomyelia (Type II)	Syringomyelia (Type III): Secondary to Spinal Cord or Intrathecal Disease	Hydromyelia (Type IV)	Hydromyelia-Central Canal (H-CCD)
**Definition and Characteristics**	Presence of a syrinx or syringobulbia visualized on MRI, involving dilation or cavitation of the spinal cord.	Syringomyelia occurs without obstruction of the foramen magnum.	Presence of a syrinx visualized on MRI, involving dilation or cavitation of the spinal cord.	Pure hydromyelia is defined as a developmental widening of the spinal cord’s central canal.	An intramedullary, centrally located, non-enhancing, slit-like cavitation (also called “idiopathic localized hydromyelia”).
**Foramen Magnum Obstruction**	Obstruction present (associated with CM1 or other obstructive lesions.	No.	No.	No.	No.
**Clinical Symptoms**	Pain, numbness, loss of sensation, and limb weakness representing cervical central cord syndrome. Headache, ataxia, and other symptoms of craniocervical syndrome.	Pain, numbness, loss of sensation, and limb weakness consistent with cervical or thoracic central myelopathy.	Pain, numbness, loss of sensation, and limb weakness consistent with thoracic or cervical central myelopathy from syringomyelia. Primary condition also produces myelopathic symptoms.	None.	None.
**Pathogenesis**	Obstruction of CSF pathways at the foramen magnum causes syrinx development and progressive enlargement of the central canal within the cervical and cervicothoracic spinal cord.	Idiopathic because syrinx is not related to a foramen magnum (or spinal) lesion.	The initiating event is known and includes tumor, trauma, post-inflammatory arachnoiditis, and other lesions obstructing CSF flow.	Congenital developmental defect in the caliber of the central canal leading to persistent filling with CSF-like fluid.	Congenital variant in which the central canal is enlarged but does not otherwise affect the spinal cord anatomy or physiology.

**Table 2:** The main epidemiological studies of syringomyelia in the post-MRI era.

Region/Country	Study Period/Year	Population	Diagnostic Criteria/Methods	Reported Prevalence Rate
**Spain** (Central provinces)	1985–1989	Eighteen-year-old male recruits	MRI	0.9 per 100,000 [43]
**New Zealand**	2003	Entire population (including Caucasians, Pacific people, and Māori)	MRI	•Entire population: 8.2:100,000 •Caucasians: 5.4:100,000 •Pacific people: 18.4:100,000 •Māori: 15.4:100,000 [18]
**Bashkortostan Republic** (Russian Federation)	2007, 2015	More than 2000 syringomyelia cases (higher prevalence in ethnic Tatars)	MRI	3.5 to 129:100,000 [44,45]
**Tatarstan** (Russian Federation)	2011	Estimated mean population/high-CM1 district	MRI	Mean prevalence: 12.6:100,000 [46]; elevated incidence in high-CM1 districts [47]
**Northwestern Italy**	2011	Regional population	MRI	4.84:100,000 (incidence: 0.82:100,000) [22]
**Japan**	2009	Nationwide population (all etiological forms)	Nationwide survey	1.94:100,000 [18]

## Data Availability

No new data were generated or analyzed in this study. Data sharing is not applicable to this article.

## Abbreviations

The following abbreviations are used in this manuscript:

CSF	Cerebrospinal fluid
CT	Computed tomography
MRI	Magnetic resonance imaging
